# Supplementary material for: Vortex-mediated spin current injection into two-dimensional superconductor NbSe2
Source: Sci Adv. 2026 Oct 21;12(43):eaeh1582. doi: 10.1126/sciadv.aeh1582 (PMC13685947; doi:10.1126/sciadv.aeh1582)
Supplement: Supplementary file 1 [file sciadv.aeh1582_sm.pdf]

Science Advances
AAAS

Supplementary Materials for

Vortex-mediated spin current injection into two-dimensional superconductor $NbSe_2$

Meng Yang *et al.*

Corresponding author: Shuo-Ying Yang, yangsy@sustech.edu.cn; Junxue Li, lijx3@sustech.edu.cn

Sci. Adv. **12**, eaeh1582 (2026)
DOI: 10.1126/sciadv.aeh1582

This PDF file includes:

Supplementary Notes S1 to S7
Figs. S1 to S8
References

Note 1. Morphological and magnetic characterization of the $LiAl_2Fe_3O_8$(LAFO) thin film

Fig. S1A shows the surface morphology of our 15 nm $LiAl_2Fe_3O_8$(LAFO) thin film used in the $NbSe_2$/LAFO devices, imaged by tapping-mode atomic force microscopy over a 5 μm × 5 μm area. The LAFO film exhibits a smooth surface free of droplets typically associated with pulsed laser deposition (PLD), with a root-mean-square roughness below 100 pm. Such an atomically flat surface ensures intimate contact between $NbSe_2$ van der Waals layer and the LAFO film, benefiting efficient spin-current injection to our devices.

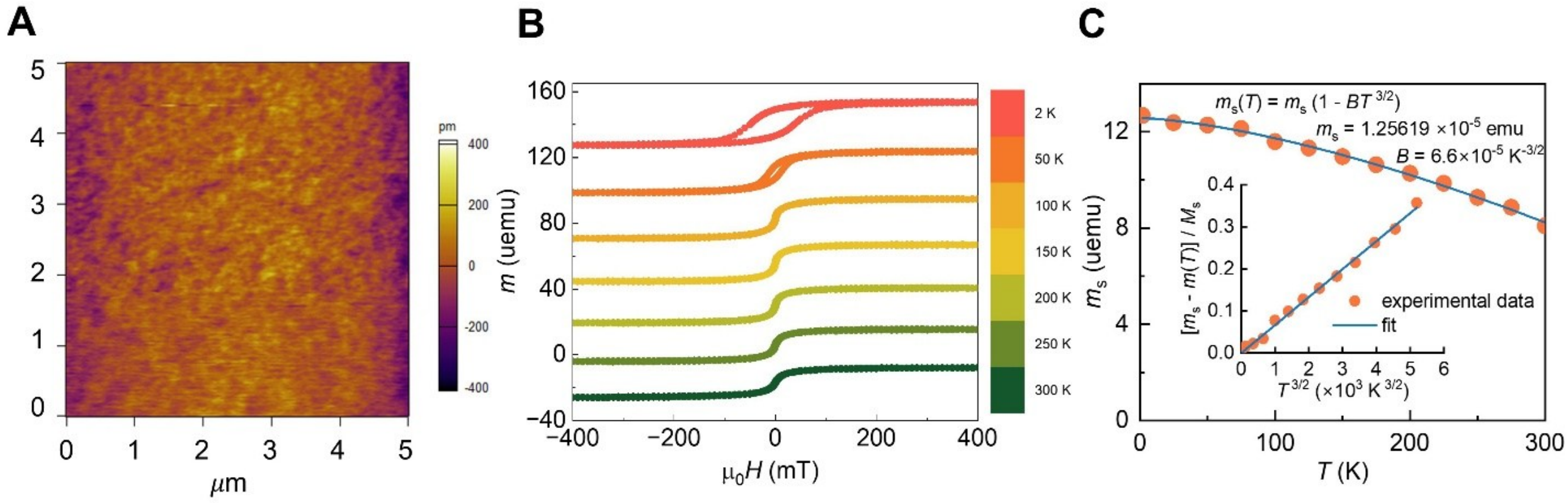

Fig. S1. Morphology and magnetic properties of a 15 nm $LiAl_2Fe_3O_8$(LAFO) film. (**A**), Atomic force microscopy image of a 15-nm-thick LAFO(001) film on $MgAl_2O_4$(001) substrate, acquired over a 5 μm × 5 μm area. The root-mean-square (rms) surface roughness is below 100 pm. (**B**), Magnetic field dependence of the magnetic moment of the LAFO film, measured by magnetic property measurement system (MPMS). The magnetic field was applied in-plane and along the [100] direction. The linear diamagnetic background originating from the $MgAl_2O_4$(001) substrate has already been subtracted here. Hysteresis loops at different temperatures are vertically offset for clarity. (**C**), Temperature dependence of the saturation magnetic moment of the 15-nm-thick LAFO(001) film. The blue curve shows a fit to $m_s(1 - BT^{3/2})$, where m_s is the saturation magnetic moment of the sample, B is a fitting parameter. The inset displays $[m_s - m(T)]/m_s$ as a function of $T^{3/2}$, exhibiting a clear linear dependence.

Fig. S1B presents the field dependence of magnetic moment for a 15-nm-thick LAFO(001) film, measured by superconducting quantum interference device (SQUID) magnetometry after subtracting the linear diamagnetic contributions from the $MgAl_2O_4$ (MAO) substrate. From 2 to 300 K, the LAFO film exhibits robust ferrimagnetic behavior. The slightly rounded hysteresis loops arise because the

magnetic field is applied along the in-plane hard axis (HA) ([100]). Even at temperatures as low as 2 K, the saturation field of the LAFO film remains below 150 mT, well below the magnetic fields at which the Nernst voltage appears in our thermoelectric experiments. This suggests that our LAFO film is uniformly magnetized during thermoelectric measurements, excluding possible contributions from domain-wall motion.

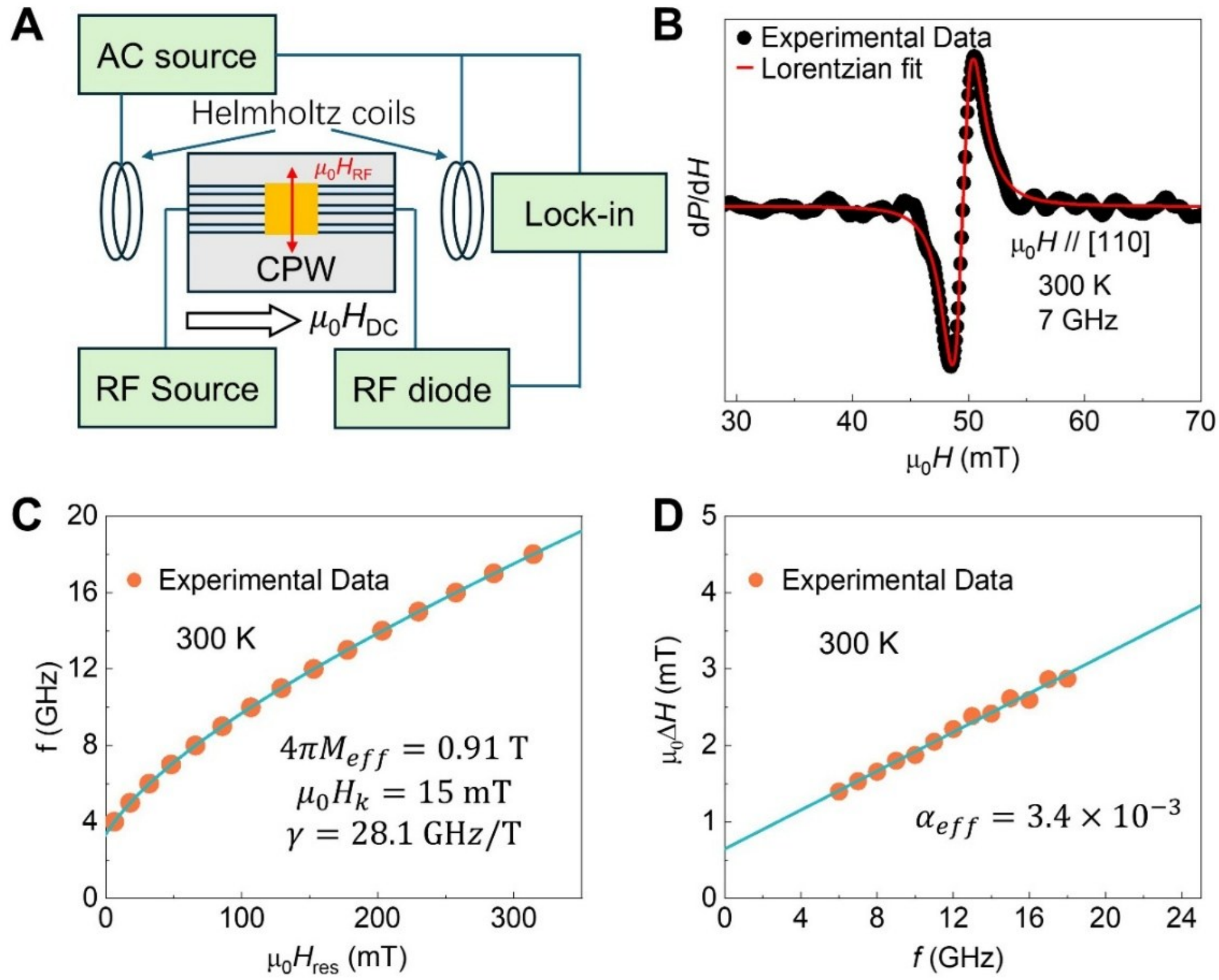

Fig. S2. Ferromagnetic resonance of a 15 nm LAFO film. (**A**), Schematic of the field-modulated ferromagnetic resonance (FMR) setup. The LAFO(15 nm) film is placed on a coplanar waveguide (CPW) and excited by microwaves, while a small a.c. field from Helmholtz coils modulates the external field; the microwave response at the modulation frequency is detected with a lock-in amplifier. (**B**), FMR line shape at 7 GHz. The solid curve represents a fit using a sum of symmetric and antisymmetric Lorentzian functions. (**C**), Frequency dependence of the resonance field. The solid line is a fit to Eq. (1). (**D**), Resonance linewidth versus frequency. The solid line is a fit to Eq. (2).

The temperature dependence of the saturated magnetic moment of our LAFO film, extracted from Fig. S1B and plotted in Fig. S1C, decreases gradually with increasing temperature but remains finite at 300 K. The data obeys a $T^{3/2}$ law, clearly indicating the softening of the magnetic moment at high

temperature is due to the spin wave excitation. Such a dependence is expected well below the Curie temperature, indicating that the Curie temperature of our LAFO film lies well above room temperature. These stable structure and magnetic properties make LAFO a favorable platform for spin-current transport experiments reported in this work.

Note 2. Magnetization dynamics of a 15 nm LAFO film

To serve as an efficient source or channel for magnon spin currents, LAFO must have low magnetic damping. Therefore, we characterized the magnetization dynamics of LAFO thin films. As shown in Fig. S2A, field-modulated ferromagnetic resonance (FMR) measurements were performed on a 15 nm LAFO(001) film. The LAFO film was placed face-down on a coplanar waveguide (CPW). A radio-frequency (RF) microwave current in the CPW generates an oscillating magnetic field (red arrows), while a static magnetic field ($\mu_0 H_{DC}$) is applied perpendicular to this RF field. When the microwave frequency and the static magnetic field satisfy the FMR condition, the film absorbs microwave and the transmitted microwave power is reduced. At the end of the CPW, the microwave power is converted into a DC voltage signal by an RF diode and detected using a lock-in amplifier. Meanwhile, an additional alternating magnetic field, produced by Helmholtz coils aligned with $\mu_0 H_{DC}$, modulates the static field. So that the lock-in working at the modulation frequency detects the derivative of the microwave absorption with respect to the static magnetic field.

Fig. S2B shows the field dependence of the differential microwave signal at 7 GHz. A single antisymmetric resonance peak appears at an external magnetic field of 50 mT, with no evidence of multiple modes arising from magnetic inhomogeneity in the sample. The resonance line shape is fitted using a combination of symmetric and antisymmetric Lorentzian functions to extract the resonance field $\mu_0 H_{res}$ and the linewidth $\mu_0 \Delta H$. The frequency dependence of $\mu_0 H_{res}$ (Fig. S2C) follows the Kittel formula:

$$f = \frac{\gamma}{2\pi}[(\mu_0 H_{res} + \mu_0 H_k)(\mu_0 H_{res} + \mu_0 H_k + 4\pi M_{eff})]^{1/2} \tag{1}$$

where γ is the gyromagnetic ratio, $\mu_0 H_k$ denotes the in-plane magnetic anisotropy field, and $4\pi M_{eff}$ represents the effective demagnetizing field. The fit yields γ = 28.1 GHz/T, $4\pi M_{eff}$ = 0.91 T, and $\mu_0 H_k$ = 15 mT, indicating that the [110] direction is the magnetic easy axis of the LAFO(001) film.

Fig. S2D shows the dependence of the resonance linewidth ($\mu_0 \Delta H$) on frequency. The Gilbert damping parameter α is extracted from a linear fit to

$$\mu_0 \Delta H = \frac{2\pi\alpha f}{\gamma} + \mu_0 \Delta H_0 \tag{2}$$

where $\mu_0 \Delta H_0$ represents the inhomogeneous broadening. From the slope, we extract the Gilbert damping $\alpha = 3.4 \times 10^{-3}$ for the LAFO film. This relatively low magnetic damping is well suited for spin-current transport and confirms that LAFO is an appropriate magnon spin current source in our experiments.

Note 3. Longitudinal spin Seebeck effect (LSSE) in Pt/LAFO heterostructure

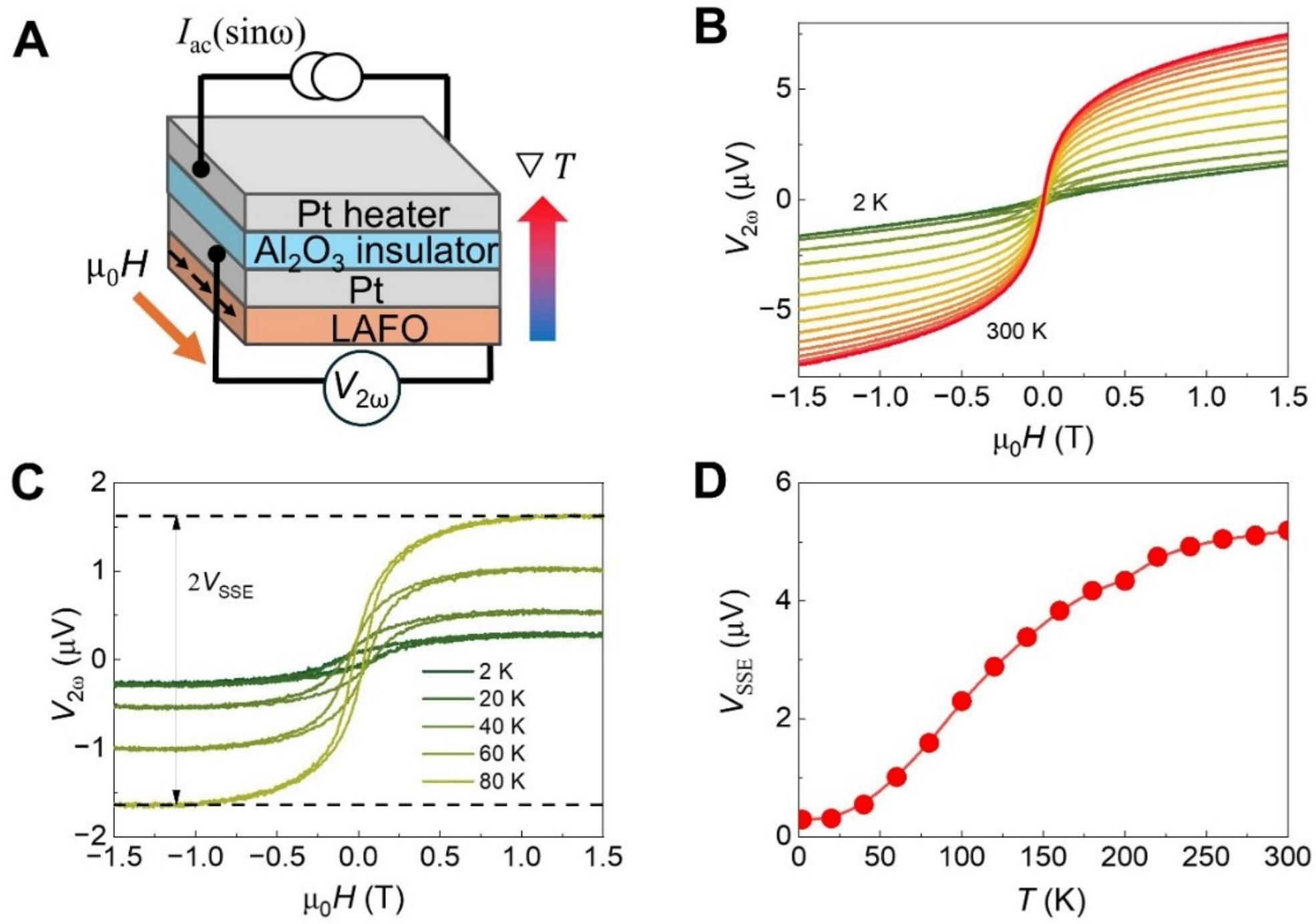

Fig. S3. Longitudinal spin Seebeck effect in a Pt/LAFO heterostructure. (**A**), Schematic of the Pt (3 nm)/LAFO (15 nm) device and measurement configuration. A low-frequency (13 Hz) alternating current is applied to the top Pt heater, the second-harmonic (2ω) voltage in the bottom Pt layer is recorded as the spin Seebeck signal while sweeping an in-plane magnetic field perpendicular to the Pt strip. (**B**), Raw second-harmonic voltage ($V_{2\omega}$) as a function of the magnetic field at selected temperatures. (**C**), Low-temperature $V_{2\omega}$–field curves after subtraction of a linear background due to the ordinary Nernst effect. (**D**), Temperature dependence of the spin Seebeck voltage magnitude V_{SSE}, as defined by the arrow in (C).

As summarized in Notes 1 and 2, the LAFO films used here combine an atomically smooth surface with low magnetic damping, making them a promising alternative to yttrium iron garnet (YIG) as a platform for spin-current transport studies (*41*, *42*). To demonstrate that LAFO can be used as an efficient spin current source, we performed the longitudinal spin Seebeck measurement in a Pt(3 nm)/LAFO(15 nm) heterostructure.

As shown in Fig. S3A, a 3-nm-thick Pt layer was first deposited on the LAFO(15 nm) film by magnetron sputtering and subsequently patterned into a Hall-bar. A 100 nm Al_2O_3 insulating layer was then deposited by atomic layer deposition (ALD) to electrically isolate the Pt/LAFO stack from the top 15 nm Pt heater, which was defined by lift-off. During the measurements, an out-of-plane temperature gradient drives thermally excited magnons and inject a spin current into the adjacent Pt layer, $\boldsymbol{J_s} = -S\nabla T$, where S is the spin Seebeck coefficient. The spin current injected into the Pt layer is converted into an electric field via the inverse spin Hall effect, $\boldsymbol{E} \propto \theta_{\rm SH}\boldsymbol{J}_{\rm s} \times \boldsymbol{\sigma}$, where $\theta_{\rm SH}$ is the spin Hall angle and $\boldsymbol{\sigma}$ denotes the spin polarization aligned with the LAFO magnetization.

Fig. S3B shows the second-harmonic voltage ($V_{2\omega}$), measured with a 5.0 mA heating current, as a function of in-plane magnetic field swept transverse to the Hall bar. The thermoelectric signal changes sign with the magnetic field reversal and follows the magnetic hysteresis loop of the LAFO film (Fig. S1B), consistent with a spin Seebeck origin. A linear background due to the ordinary Nernst effect in Pt is removed before extracting the LSSE amplitude (Fig. S3C). As summarized in Fig. S3D, the LSSE magnitude decreases monotonically from 300 K to 2 K, consistent with the second law of thermal dynamics.

Note 4. Optical images of the devices

Fig. S4A and S4B show optical images of the $NbSe_2/SiO_2/Si$ and $NbSe_2/LiAl_2Fe_3O_8$(LAFO) devices used in the main text, respectively. The Pt top heater and $NbSe_2$ layer are electrically isolated from each other by an h-BN spacer. The resistance between them exceeds 10^9 Ω, effectively excluding current-induced artifacts arising from electrical leakage. The voltage polarity is defined as shown in the figure.

In the $NbSe_2/SiO_2/Si$ device, a negative thermoelectric voltage is observed under a magnetic field

along $+y$ direction, consistent with the vortex Nernst polarity given by $E = B \times v_T$. By contrast, the $NbSe_2$/LAFO device shows a reversed thermoelectric voltage polarity, dominated by the contribution $E = B \times v_s$ from spin-driven vortex motion. The observed polarity of the thermoelectric voltage in $NbSe_2$/LAFO is therefore consistent with the proposed spin-vorticity transmutation mechanism.

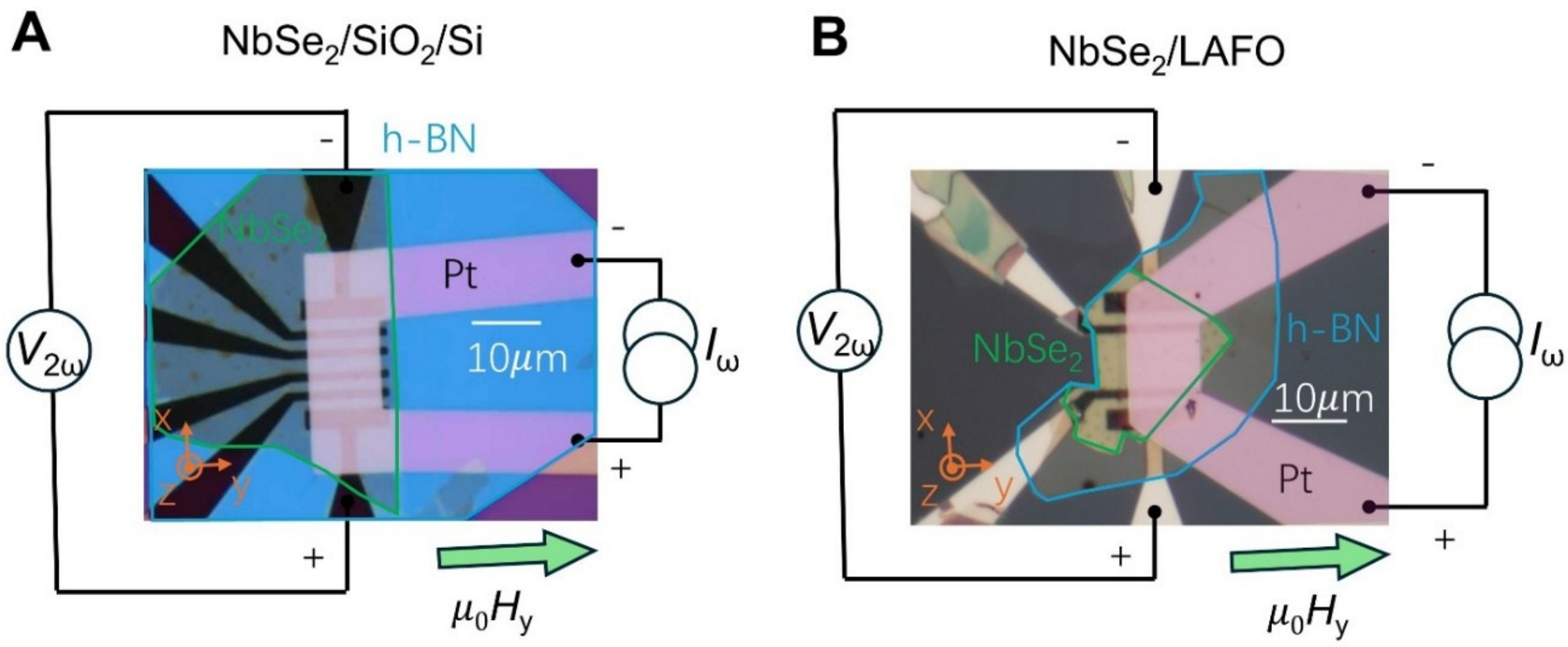

Fig. S4. Optical images of the $NbSe_2/SiO_2$/Si (A) and $NbSe_2/LiAl_2Fe_3O_8$(LAFO) (B) devices. The $NbSe_2$ layer (green), h-BN encapsulation (blue) and Pt top heater (purple) are highlighted in false color. An alternating current is applied to the Pt heater to generate an out-of-plane temperature gradient, and the second-harmonic (2ω) voltage in the $NbSe_2$ layer is probed to quantify the thermoelectric response.

Note 5. Thermoelectric response in different samples

The thermoelectric voltage signals are reproducible across multiple samples. Fig. S5A and S5B show optical images of device 3 with an $NbSe_2$(11 nm)/LAFO(15 nm) bilayer, and device 4 with an $NbSe_2$(24 nm)/LAFO(15 nm) bilayer, respectively. Fig. S5D and S5E present the thermoelectric voltages, measured in the same configuration as in the main text. In both devices, the voltage polarity matches that of the $NbSe_2$/LAFO sample discussed in the main text, suggesting that the observed spin-vortex transmutation signals are highly reproducible and robust. In device 3, a relatively large heating current (2.5 mA) was used during the measurement, bringing the effective sample temperature close to the superconducting transition. An abrupt change in the thermoelectric voltage at low magnetic field has been observed in Fig. S5D, which is constrained in a very narrow temperature window and shows no hysteresis, therefore we attribute it to vortex creeping in weak magnetic field.

To further verify the sign of the vortex Nernst signal in $NbSe_2$, we fabricated an additional $NbSe_2$ single-layer device (device 5) on a non-magnetic $MgAl_2O_4$(MAO) substrate (Fig. S5C). The vortex Nernst signal of a $NbSe_2$(12 nm)/MAO system (Fig. S5F) exhibits the same polarity as that of the $NbSe_2$(15 nm)/SiO_2/Si sample in Fig. 2, consistent with expectations for a VNE driven by an out-of-plane temperature gradient. The oscillatory thermoelectric signal observed at low magnetic field in Fig. S5F may also stem from vortex creep, which is sensitive to defect landscape in the sample. Overall, the VNE polarity of $NbSe_2$ is reproducible on both SiO_2/Si and MAO substrates.

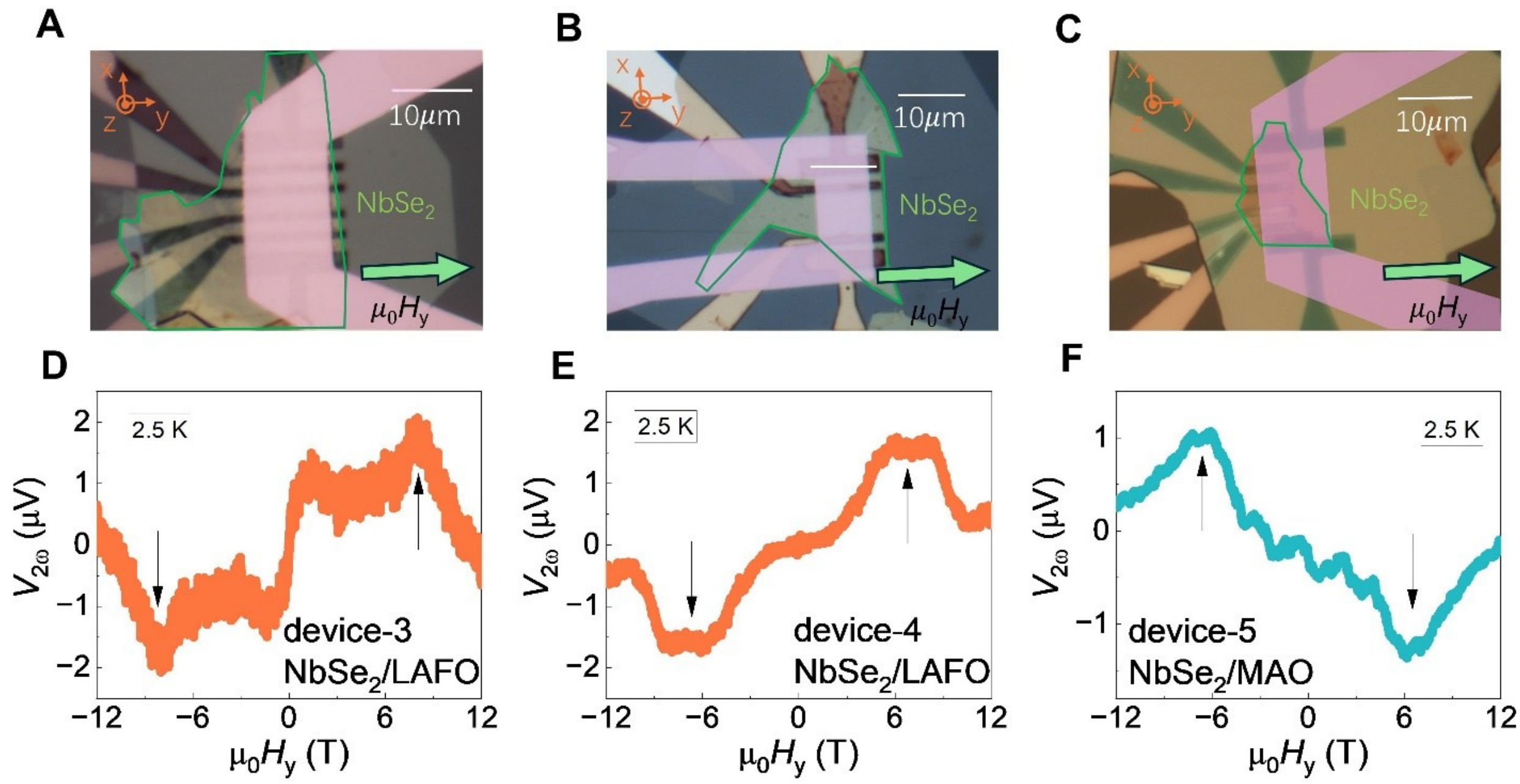

Fig. S5. Device images and thermoelectric signals in different $NbSe_2$ heterostructures at 2.5 K. (**A-C**) Optical images of device 3 ($NbSe_2$(11 nm)/LAFO(15 nm)) (**A**), device 4 ($NbSe_2$(24 nm)/LAFO(15 nm)) (**B**), and device 5 ($NbSe_2$(12 nm)/$MgAl_2O_4$(001)) (**C**). In all devices, the top heater is made of 15 nm Pt stripe. **(D-F)** Thermoelectric voltage as a function of in-plane magnetic field along y-direction for devices 3 (**D**), 4 (**E**) and 5 (**F**) at 2.5 K. For devices 3 and 4, the Nernst signal is obtained by antisymmetrizing the raw field-dependent data.

Note 6. Lower critical magnetic field of the $NbSe_2$ single crystal

The magnetic field starts to penetrate the superconductor once it exceeds the lower critical magnetic field, μ_0H_{c1}. We determine $\mu_0 H_{c1}$ for the $NbSe_2$ single crystal from field-dependent magnetic moment (*m*) measurements.

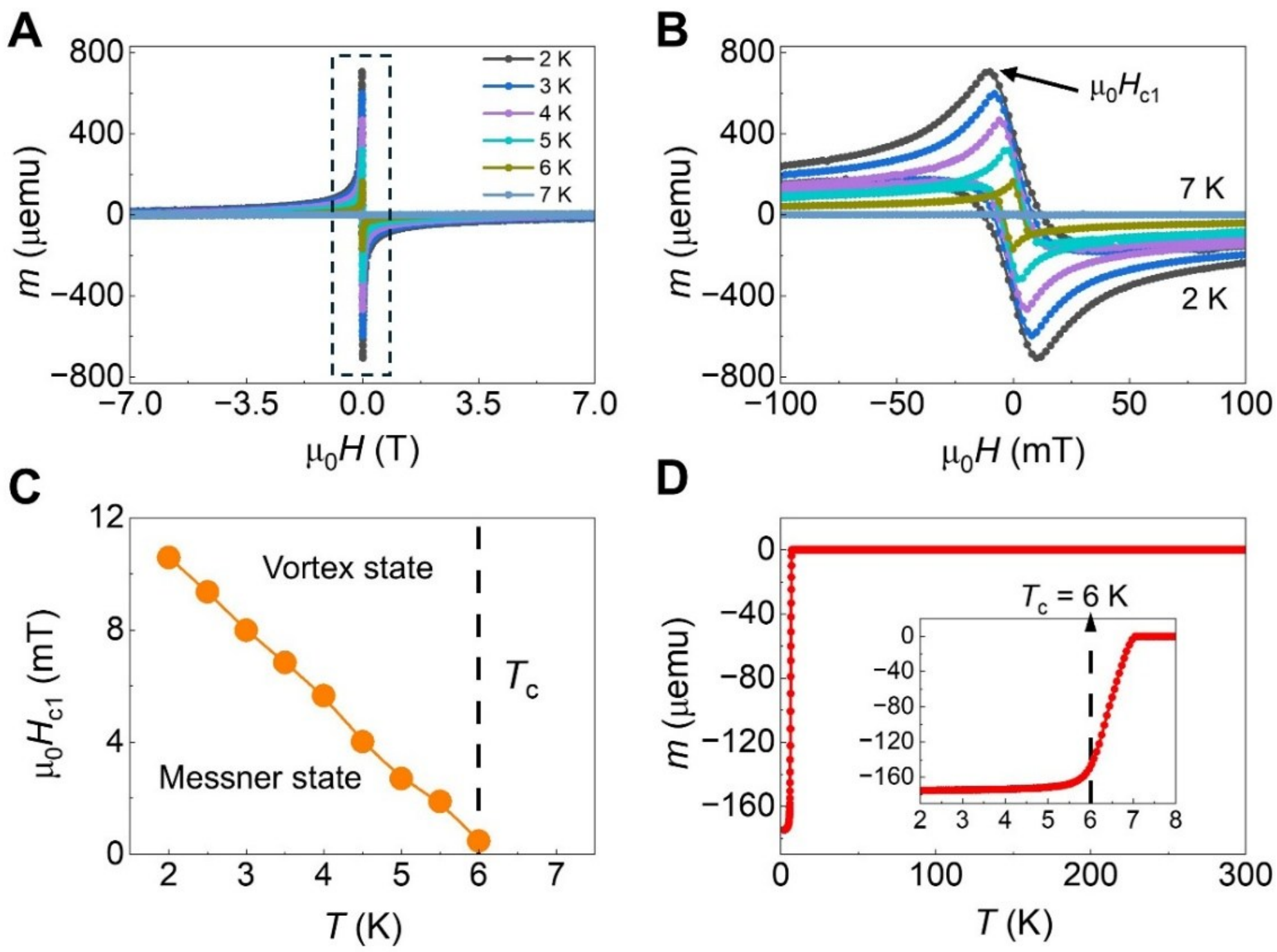

Fig. S6. Determination of the lower critical magnetic field $\mu_0 H_{c1}$ of the $NbSe_2$ single crystal. (**A**), Magnetic moment (*m*) of the $NbSe_2$ single crystal as a function of an in-plane magnetic field at selected temperatures from 2 K to 7 K. (**B**), Enlarged view of the low-field region of the *m*-*H* curves in (A). The lower critical magnetic field $\mu_0 H_{c1}$ of $NbSe_2$ is indicated by an arrow. (**C**), Temperature dependence of $\mu_0 H_{c1}$ extracted from the *m*-*H* curves, shown in (B). (**D**), Temperature dependence of the magnetic moment under an in-plane magnetic field of 1 mT after zero-field cooling. The inset shows an enlarged view of the low-temperature region. The critical temperature of the $NbSe_2$ crystal is 6.0 K.

As shown in Fig. S6A and S6B, the sample remains in the Meissner state when the in-plane magnetic field is below $\mu_0 H_{c1}$, and the diamagnetic response yields a linear m-H relation. As the magnetic field exceeds $\mu_0 H_{c1}$, the vortices enter the $NbSe_2$ crystal, weakening the diamagnetic response and causing the magnetic moment to increase with the magnetic field. We identify $\mu_0 H_{c1}$ as the field at which the m-H curve shows a peak or dip (Fig. S6B).

The extracted $\mu_0 H_{c1}$ at different temperatures is summarized in Fig. S6C. $\mu_0 H_{c1}$ decreases with increasing temperature and becomes zero around 6 K, consistent with our independent measurement of the critical temperature (Fig. S6D). Over the measured temperature range, the in-plane lower critical field of the $NbSe_2$ single crystal remains below 12 mT, in good agreement with previous reports (*55*).

The thickness of the exfoliated $NbSe_2$ layer in our devices is above 15 nm, and its critical temperature (T_c) is very close to that of the bulk crystal, so we assume the $\mu_0 H_{c1}$ of our exfoliated $NbSe_2$ layer is the same as that of the bulk crystal measured here.

Notably, the $\mu_0 H_{c1}$ of the $NbSe_2$ crystal is substantially lower than the vortex-lattice-melting field $\mu_0 H_{melt}$ where the thermoelectric signals emerge, ruling out contributions from both the Meissner state and the vortex-solid phase to the observed thermoelectric signal. Together with the superconducting phase diagram, these results support the interpretation that the thermoelectric signal arises predominantly from the vortex-liquid regime.

Note 7. Thermoelectric response under an inverted temperature gradient

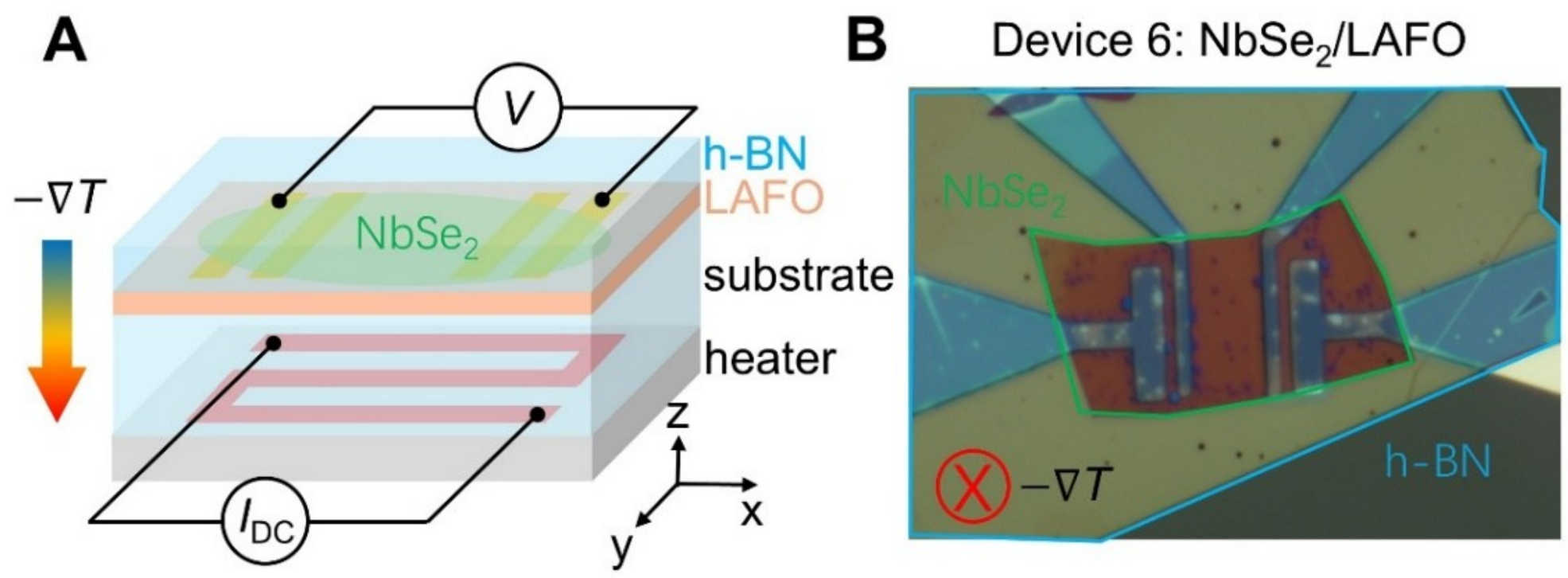

Fig. S7. Structure and optical image of the reference $NbSe_2$/LAFO device measured under an inverted temperature gradient. (**A**), Schematic of the bottom-heating measurement geometry. A direct current applied to the bottom heater generates an inverted temperature gradient ($-\nabla T$). (**B**), Optical image of the reference device 6 [$NbSe_2$(17 nm)/LAFO(15 nm)].

To examine how the thermoelectric signal depends on the direction of the temperature gradient, we carried out thermoelectric measurements on a reference $NbSe_2$/LAFO bilayer device (device 6) under an inverted temperature gradient, $-\nabla T$. As shown in Fig. S7A, a serpentine Pt heater (15 nm thick) was first fabricated on an MAO(001) substrate using standard lift-off procedure. We then fabricated the $NbSe_2$/LAFO bilayer device (without a top heater) on a separate substrate and attached this device, with its substrate, onto the heater substrate using GE varnish. The $NbSe_2$ flake was encapsulated with a thin h-BN layer to prevent degradation. During the measurements, an inverted temperature gradient ($-\nabla T$) is produced by a direct current in the bottom heater due to Joule heating,

and the open-circuit voltage is probed in $NbSe_2$ while sweeping a magnetic field along the y-direction.

We first characterized the superconducting property of the reference device, whose optical image is shown in Fig. S7B. Fig. S8A shows the temperature dependence of the four-terminal resistance of the $NbSe_2$/LAFO bilayer device 6. It exhibits a sharp superconducting transition, with Tc comparable to that of bulk $NbSe_2$. When a 1.0 mA direct current is applied to the bottom heater, the critical temperature of the $NbSe_2$ layer decreases, confirming that the bottom heater is effectively generating a temperature gradient.

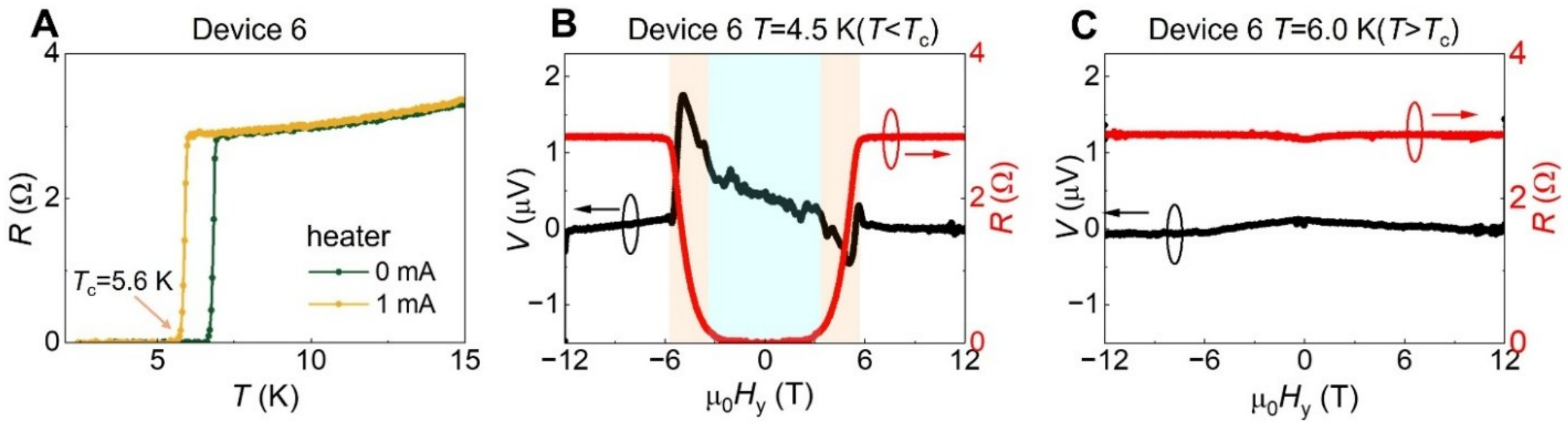

Fig. S8. Thermoelectric measurements under an inverted temperature gradient. (**A**), Four-terminal resistance as a function of temperature for device 6, measured without heating (heater off) and with a 1.0 mA DC current applied to the bottom heater. (**B**), Thermoelectric voltage as a function of the in-plane magnetic field $\mu_0 H_y$ for device 6, measured below the superconducting transition temperature, with a 1.0 mA bottom heating current. The corresponding field-dependent four-terminal resistance at the heater-on state is also shown. The Meissner and vortex-solid regimes are shaded in light blue, and the vortex-liquid regime is shaded in orange. (**C**), Thermoelectric voltage and four-terminal resistance as a function of the magnetic field, measured above the superconducting transition temperature, with a 1.0 mA bottom heating current.

Fig. S8B presents the thermoelectric voltage as a function of the in-plane magnetic field $\mu_0 H_y$, measured below the superconducting transition temperature T_c. The corresponding four-terminal resistance measured under a 1 mA heating current is also plotted to track the superconducting state. The finite thermoelectric signal that shows up around zero field can be ascribed to the creeping of the vortices in the vortex-solid regime (light blue region). A pronounced thermoelectric signal emerges around the $\mu_0 H_{c2}$, consistent with the spin-driven vortex flow (SDVF) in the vortex-liquid regime (orange region). The thermoelectric signal vanishes in the normal state ($T>T_c$) because the

superconducting vortex state no longer exists (Fig. S8C). It is worth noting that the thermoelectric signal due to SDVF under the inverted temperature gradient ($-\nabla T$) exhibits an opposite sign to that in the LAFO/$NbSe_2$ devices with a top heater ($+\nabla T$) (Figs. 2 and 3 in the main text).

REFERENCES

1. W. Han, S. Maekawa, X.-C. Xie, Spin current as a probe of quantum materials. *Nat. Mater.* **19**, 139–152 (2020).

2. J. Linder, J. W. A. Robinson, Superconducting spintronics. *Nat. Phys.* **11**, 307–315 (2015).

3. R. S. Keizer, S. T. B. Goennenwein, T. M. Klapwijk, G. Miao, G. Xiao, A. Gupta, A spin triplet supercurrent through the half-metallic ferromagnet CrO_2. *Nature* **439**, 825–827 (2006).

4. D. Sanchez-Manzano, S. Mesoraca, F. A. Cuellar, M. Cabero, V. Rouco, G. Orfila, X. Palermo, A. Balan, L. Marcano, A. Sander, M. Rocci, J. Garcia-Barriocanal, F. Gallego, J. Tornos, A. Rivera, F. Mompean, M. Garcia-Hernandez, J. M. Gonzalez-Calbet, C. Leon, S. Valencia, C. Feuillet-Palma, N. Bergeal, A. I. Buzdin, J. Lesueur, J. E. Villegas, J. Santamaria, Extremely long-range, high-temperature Josephson coupling across a half-metallic ferromagnet. *Nat. Mater.* **21**, 188–194 (2022).

5. K.-R. Jeon, C. Ciccarelli, A. J. Ferguson, H. Kurebayashi, L. F. Cohen, X. Montiel, M. Eschrig, J. W. A. Robinson, M. G. Blamire, Enhanced spin pumping into superconductors provides evidence for superconducting pure spin currents. *Nat. Mater.* **17**, 499–503 (2018).

6. L. P. Gor'kov, E. I. Rashba, Superconducting 2D system with lifted spin degeneracy: Mixed singlet-triplet state. *Phys. Rev. Lett.* **87**, 037004 (2001).

7. F. S. Bergeret, I. V. Tokatly, Spin-orbit coupling as a source of long-range triplet proximity effect in superconductor-ferromagnet hybrid structures. *Phys. Rev. B* **89**, 134517 (2014).

8. F. Hübler, M. J. Wolf, D. Beckmann, H. v. Löhneysen, Long-range spin-polarized quasiparticle transport in mesoscopic Al superconductors with a Zeeman splitting. *Phys. Rev. Lett.* **109**, 207001 (2012).

9. C. H. L. Quay, D. Chevallier, C. Bena, M. Aprili, Spin imbalance and spin-charge separation in a mesoscopic superconductor. *Nat. Phys.* **9**, 84–88 (2013).

10. H. L. Zhao, S. Hershfield, Tunneling, relaxation of spin-polarized quasiparticles, and spin-charge separation in superconductors. *Phys. Rev. B* **52**, 3632–3638 (1995).

11. T. Wakamura, H. Akaike, Y. Omori, Y. Niimi, S. Takahashi, A. Fujimaki, S. Maekawa, Y. Otani, Quasiparticle-mediated spin Hall effect in a superconductor. *Nat. Mater.* **14**, 675–678 (2015).

12. S. Takahashi, S. Maekawa, Hall effect induced by a spin-polarized current in superconductors. *Phys. Rev. Lett.* **88**, 116601 (2002).

13. T. Wakamura, N. Hasegawa, K. Ohnishi, Y. Niimi, Y. Otani, Spin injection into a superconductor with strong spin-orbit coupling. *Phys. Rev. Lett.* **112**, 036602 (2014).

14. A. Llordés, A. Palau, J. Gázquez, M. Coll, R. Vlad, A. Pomar, J. Arbiol, R. Guzmán, S. Ye, V. Rouco, F. Sandiumenge, S. Ricart, T. Puig, M. Varela, D. Chateigner, J. Vanacken, J. Gutiérrez, V. Moshchalkov, G. Deutscher, C. Magen, X. Obradors, Nanoscale strain-induced pair suppression as a vortex-pinning mechanism in high-temperature superconductors. *Nat. Mater.* **11**, 329–336 (2012).

15. T. Puig, J. Gutierrez, X. Obradors, Impact of high growth rates on the microstructure and vortex pinning of high-temperature superconducting coated conductors. *Nat. Rev. Phys.* **6**, 132–148 (2024).

16. M. Borst, P. H. Vree, A. Lowther, A. Teepe, S. Kurdi, I. Bertelli, B. G. Simon, Y. M. Blanter, T. van der Sar, Observation and control of hybrid spin-wave–Meissner-current transport modes. *Science* **382**, 430–434 (2023).

17. O. V. Dobrovolskiy, R. Sachser, T. Brächer, T. Böttcher, V. V. Kruglyak, R. V. Vovk, V. A. Shklovskij, M. Huth, B. Hillebrands, A. V. Chumak, Magnon–fluxon interaction in a ferromagnet/superconductor heterostructure. *Nat. Phys.* **15**, 477–482 (2019).

18. O. V. Dobrovolskiy, Q. Wang, D. Y. Vodolazov, R. Sachser, M. Huth, S. Knauer, A. I. Buzdin, Moving Abrikosov vortex lattices generate sub-40-nm magnons. *Nat. Nanotechnol.* **20**, 1764–1770 (2025).

19. S. K. Kim, R. Myers, Y. Tserkovnyak, Nonlocal spin transport mediated by a vortex liquid in superconductors. *Phys. Rev. Lett.* **121**, 187203 (2018).

20. T. Taira, Y. Kato, M. Ichioka, H. Adachi, Spin Hall effect generated by fluctuating vortices in type-II superconductors. *Phys. Rev. B* **103**, 134417 (2021).

21. H. Adachi, Y. Kato, J.-i. Ohe, M. Ichioka, Time-dependent Ginzburg-Landau theory of the vortex spin Hall effect. *Phys. Rev. B* **109**, 174503 (2024).

22. A. Vargunin, M. Silaev, Flux flow spin Hall effect in type-II superconductors with spin-splitting field. *Sci. Rep.* **9**, 5914 (2019).

23. P. W. Anderson, Two new vortex liquids. *Nat. Phys.* **3**, 160–162 (2007).

24. L. Li, J. G. Checkelsky, S. Komiya, Y. Ando, N. P. Ong, Low-temperature vortex liquid in $La_{2-x}Sr_xCuO_4$. *Nat. Phys.* **3**, 311–314 (2007).

25. K.-i. Uchida, H. Adachi, T. Ota, H. Nakayama, S. Maekawa, E. Saitoh, Observation of longitudinal spin-Seebeck effect in magnetic insulators. *Appl. Phys. Lett.* **97**, 172505 (2010).

26. J. Li, H. T. Simensen, D. Reitz, Q. Sun, W. Yuan, C. Li, Y. Tserkovnyak, A. Brataas, J. Shi, Observation of magnon polarons in a uniaxial antiferromagnetic insulator. *Phys. Rev. Lett.* **125**, 217201 (2020).

27. A. Pourret, P. Spathis, H. Aubin, K. Behnia, Nernst effect as a probe of superconducting fluctuations in disordered thin films. *New J. Phys.* **11**, 055071 (2009).

28. T. Song, Y. Jia, G. Yu, Y. Tang, P. Wang, R. Singha, X. Gui, A. J. Uzan-Narovlansky, M. Onyszczak, K. Watanabe, T. Taniguchi, R. J. Cava, L. M. Schoop, N. P. Ong, S. Wu, Unconventional superconducting quantum criticality in monolayer WTe_2. *Nat. Phys.* **20**, 269–274 (2024).

29. C. W. Rischau, Y. Li, B. Fauqué, H. Inoue, M. Kim, C. Bell, H. Y. Hwang, A. Kapitulnik, K. Behnia, Universal bound to the amplitude of the vortex Nernst signal in superconductors. *Phys. Rev. Lett.* **126**, 077001 (2021).

30. K. Behnia, H. Aubin, Nernst effect in metals and superconductors: A review of concepts and experiments. *Rep. Prog. Phys.* **79**, 046502 (2016).

31. Y. Wang, L. Li, N. P. Ong, Nernst effect in high-T_c superconductors. *Phys. Rev. B* **73**, 024510 (2006).

32. P. W. Anderson, Y. B. Kim, Hard superconductivity: Theory of the motion of Abrikosov flux lines. *Rev. Mod. Phys.* **36**, 39–43 (1964).

33. J. Bardeen, M. J. Stephen, Theory of the motion of vortices in superconductors. *Phys. Rev.* **140**, A1197–A1207 (1965).

34. R. P. Huebener, A. Seher, Nernst effect and flux flow in superconductors. I. Niobium. *Phys. Rev.* **181**, 701–709 (1969).

35. X. Xi, L. Zhao, Z. Wang, H. Berger, L. Forró, J. Shan, K. F. Mak, Strongly enhanced charge-density-wave order in monolayer $NbSe_2$. *Nat. Nanotechnol.* **10**, 765–769 (2015).

36. X. Xi, Z. Wang, W. Zhao, J.-H. Park, K. T. Law, H. Berger, L. Forró, J. Shan, K. F. Mak, Ising pairing in superconducting $NbSe_2$ atomic layers. *Nat. Phys.* **12**, 139–143 (2016).

37. X. Xi, H. Berger, L. Forró, J. Shan, K. F. Mak, Gate tuning of electronic phase transitions in two-dimensional $NbSe_2$. *Phys. Rev. Lett.* **117**, 106801 (2016).

38. P. Wan, O. Zheliuk, N. F. Q. Yuan, X. Peng, L. Zhang, M. Liang, U. Zeitler, S. Wiedmann, N. E. Hussey, T. T. M. Palstra, J. Ye, Orbital Fulde–Ferrell–Larkin–Ovchinnikov state in an Ising superconductor. *Nature* **619**, 46–51 (2023).

39. C.-w. Cho, T. T. Lortz, K. T. Lo, C. Y. Ng, S. H. Chui, A. R. Allan, M. Abdel-Hafiez, J. Park, B. Cho, K. Park, N. F. Q. Yuan, R. Lortz, Evidence for orbital Fulde-Ferrell-Larkin-Ovchinnikov state in the bulk limit of 2H-$NbSe_2$. *Nat. Commun.* **16**, 7599 (2025).

40. H. Ren, X. Y. Zheng, S. Channa, G. Wu, D. A. O'Mahoney, Y. Suzuki, A. D. Kent, Hybrid spin Hall nano-oscillators based on ferromagnetic metal/ferrimagnetic insulator heterostructures. *Nat. Commun.* **14**, 1406 (2023).

41. X. Y. Zheng, S. Channa, L. J. Riddiford, J. J. Wisser, K. Mahalingam, C. T. Bowers, M. E. McConney, A. T. N'Diaye, A. Vailionis, E. Cogulu, H. Ren, Z. Galazka, A. D. Kent, Y. Suzuki, Ultra-thin lithium aluminate spinel ferrite films with perpendicular magnetic anisotropy and low damping. *Nat. Commun.* **14**, 4918 (2023).

42. D. O'Mahoney, S. Channa, X. Y. Zheng, A. Vailionis, P. Shafer, A. T. N'Diaye, C. Klewe, Y. Suzuki, Aluminum substitution in low damping epitaxial lithium ferrite films. *Appl. Phys. Lett.* **123**, 172405 (2023).

43. T. Miyasato, N. Abe, T. Fujii, A. Asamitsu, S. Onoda, Y. Onose, N. Nagaosa, Y. Tokura, Crossover behavior of the anomalous Hall effect and anomalous Nernst effect in itinerant ferromagnets. *Phys. Rev. Lett.* **99**, 086602 (2007).

44. Y. Pu, D. Chiba, F. Matsukura, H. Ohno, J. Shi, Mott relation for anomalous Hall and Nernst effects in $Ga_{1-x}Mn_xAs$ ferromagnetic semiconductors. *Phys. Rev. Lett.* **101**, 117208 (2008).

45. T. C. Chuang, P. L. Su, P. H. Wu, S. Y. Huang, Enhancement of the anomalous Nernst effect in ferromagnetic thin films. *Phys. Rev. B* **96**, 174406 (2017).

46. H. Sharma, Z. Wen, M. Mizuguchi, Spin Seebeck effect mediated reversal of vortex-Nernst effect in superconductor-ferromagnet bilayers. *Sci. Rep.* **13**, 4425 (2023).

47. G. Blatter, M. V. Feigel'man, V. B. Geshkenbein, A. I. Larkin, V. M. Vinokur, Vortices in high-temperature superconductors. *Rev. Mod. Phys.* **66**, 1125–1388 (1994).

48. Y. Shiomi, J. Lustikova, E. Saitoh, Oscillatory Nernst effect in Pt|ferrite|cuprate-superconductor trilayer films. *Sci. Rep.* **7**, 5358 (2017).

49. R. Bel, K. Behnia, H. Berger, Ambipolar Nernst effect in $NbSe_2$. *Phys. Rev. Lett.* **91**, 066602 (2003).

50. R. Daou, J. Chang, D. LeBoeuf, O. Cyr-Choinière, F. Laliberté, N. Doiron-Leyraud, B. J. Ramshaw, R. Liang, D. A. Bonn, W. N. Hardy, L. Taillefer, Broken rotational symmetry in the pseudogap phase of a high-T_c superconductor. *Nature* **463**, 519–522 (2010).

51. M. Umeda, Y. Shiomi, T. Kikkawa, T. Niizeki, J. Lustikova, S. Takahashi, E. Saitoh, Spin-current coherence peak in superconductor/magnet junctions. *Appl. Phys. Lett.* **112**, 232601 (2018).

52. K.-R. Jeon, J.-C. Jeon, X. Zhou, A. Migliorini, J. Yoon, S. S. P. Parkin, Giant transition-state quasiparticle spin-Hall effect in an exchange-spin-split superconductor detected by nonlocal magnon spin transport. *ACS Nano* **14**, 15874–15883 (2020).

53. L. Nádvorník, O. Gueckstock, L. Braun, C. Niu, J. Gräfe, G. Richter, G. Schütz, H. Takagi, M. Zeer, T. S. Seifert, P. Kubaščík, A. K. Pandeya, A. Anane, H. Yang, A. Bedoya-Pinto, S. S. P. Parkin, M. Wolf, Y. Mokrousov, H. Nakamura, T. Kampfrath, Terahertz spin-to-charge current conversion in stacks of ferromagnets and the transition-metal dichalcogenide $NbSe_2$. *Adv. Mater. Interfaces* **9**, 2201675 (2022).

54. N. Vlietstra, J. Shan, V. Castel, B. J. van Wees, J. Ben Youssef, Spin-Hall magnetoresistance in platinum on yttrium iron garnet: Dependence on platinum thickness and in-plane/out-of-plane magnetization. *Phys. Rev. B* **87**, 184421 (2013).

55. J. del Valle, A. Gomez, E. M. Gonzalez, S. Manas-Valero, E. Coronado, J. L. Vicent, Force-free state in a superconducting single crystal and angle-dependent vortex helical instability. *Phys. Rev. B* **95**, 224510 (2017).
